# The Urinary Phosphate to Serum Fibroblast Growth Factor 23 Ratio, Deemed the Nephron Index, Is a Useful Clinical Index for Early Stage Chronic Kidney Disease in Patients with Type 2 Diabetes: An Observational Pilot Study

**DOI:** 10.1155/2018/7530923

**Published:** 2018-09-02

**Authors:** Hodaka Yamada, Makoto Kuro-o, Shunsuke Funazaki, San-e Ishikawa, Masafumi Kakei, Kazuo Hara

**Affiliations:** ^1^Department of Medicine, Division of Endocrinology and Metabolism, Jichi Medical University Saitama Medical Center, 1-847 Amanuma-cho, Omiya-ku, Saitama 330-8503, Japan; ^2^Center for Molecular Medicine, Jichi Medical University, 3311-1 Yakushiji Shimotsuke, Tochigi 329-0498, Japan; ^3^Division of Endocrinology and Metabolism, International University of Health and Welfare Hospital, 537-3 Iguchi, Nasushiobara, Tochigi 329-2763, Japan

## Abstract

Renal function decline is associated with progressive type 2 diabetes mellitus, which causes mineral and bone disorders. In the present study, we defined the ratio of urinary phosphate excretion (mg/day) to serum fibroblast growth factor 23 as the nephron index. We examined changes in the nephron index in type 2 diabetes patients with early stage chronic kidney disease (stages 1–3), enrolling 15 patients and retrospectively analysing the follow-up data. After follow-up at 5.4 years, we observed no significant changes in the estimated glomerular filtration rate; the nephron index, however, was significantly reduced between the baseline and the follow-up. We propose that the nephron index may be potentially useful as a biomarker for monitoring the decline of renal function in the early stages of diabetic chronic kidney disease patients.

## 1. Introduction

Several biomarkers relevant to bone and mineral metabolism in chronic kidney disease (CKD)—for which type 2 diabetes mellitus (T2DM) is an important risk—are associated with cardiovascular complications. Among them, serum levels of fibroblast growth factor 23 (FGF23) have been identified, from as early as stage 2 CKD, as one of the earliest biomarkers that start increasing, and correlate with cardiac hypertrophy, heart failure, and all-cause mortality [1, 2].

Following phosphate intake, the bone secretes the peptide hormone FGF23, which acts on the kidney to induce urinary phosphate excretion, thereby maintaining the phosphate balance. However, for several hours after oral phosphate administration, T2DM patients reportedly showed higher serum phosphate and lower serum FGF23 levels than nondiabetes patients, even when there was no difference in their renal function [3]. The measurement of serum FGF23 levels alone—as suggested by these results—may not be sufficient for the early detection of renal dysfunction in CKD patients with differing pathogeneses.

FGF23 exerts phosphaturic activity through its ability to suppress phosphate resorption in the renal proximal tubule. FGF23 is thus thought to correlate with phosphate excretion per nephron. Namely,

(1) phosphate  excretion  per  nephron=urinary  phosphate  excretionnephron  number∝FGF23

(2) ∴nephron  number∝urinary  phosphate  excretionFGF23≡nephronindex

We hypothesise the ratio of urinary phosphate excretion (mg/day) to serum FGF23 to serve as an index correlating with the nephron number, which we define as the nephron index [4, 5].

In a recent cross-sectional study, we reported the nephron index to be decreased in T2DM patients with stages 1–3 CKD and associated with macrovascular complications [6]. In the present pilot study, during longitudinal follow-up, we examined changes in the nephron index in T2DM patients.

## 2. Material and Methods

We enrolled 18 patients with T2DM who had been admitted twice to Jichi Medical University Saitama Medical Center for diabetes control between October 2006 and January 2017 depending on the 2-week diabetes education program. The 24-hour urine collections were performed 10 days after the hospitalization. All the patients had an estimated glomerular filtration rate (eGFR) greater than 45 mL/min/1.73 m^2^. Because 3 patients had incomplete urine collection records, we did not consider them for this study, and finally we analysed the present data for 15 patients (CKD categories, number of patients: G1, 4; G2, 8; G3a, 3; A1, 11; and A2, 4). Daily phosphate intake had been managed between 600 and 700 mg/day by a nutritionist. We used fasting blood samples to measure 24 h urinary phosphate excretion (mg/day) and other mineral and metabolic parameters. We collected the clinical parameters based on medical records and laboratory data. Intact FGF23 had been measured using an enzyme-linked immunosorbent assay (ELISA) (Kainos, Tokyo, Japan) in our previous study [6]. We compared the parameters from baseline (the first admission) with those after follow-up (the second admission). Other variables had been measured at a central laboratory section of the Jichi Medical University Saitama Medical Center. Renal function was determined with calculation of eGFR using the modification of diet in renal disease equation revised for the Japanese population by the Japanese Society of Nephrology as follows: eGFR (mL/min/1.73 m^2^) = 194 × serum creatinine (mg/dL)^−1.094^× age^−0.287^× 0.739 (if female). Baseline and follow-up eGFR was calculated by blood test for each hospitalization. We compared the data relating to the two dates using a paired* t* test. Data are expressed as the mean ± standard deviation or median with interquartile range. We considered a* p* value < 0.05 to be statistically significant. We performed all analyses using EZR (Saitama Medical Center, Jichi Medical University), a graphical user interface for R (the R Foundation for Statistical Computing, ver. 2.13.0) and a modified version of the R Commander (ver. 1.6-3), with additional frequently used biostatistical functions [7].

This study was approved by the Ethics Committee at Jichi Medical University Saitama Medical Center (No. S17-007) and performed in compliance with the Declaration of Helsinki.

## 3. Results

**Figure 1:**
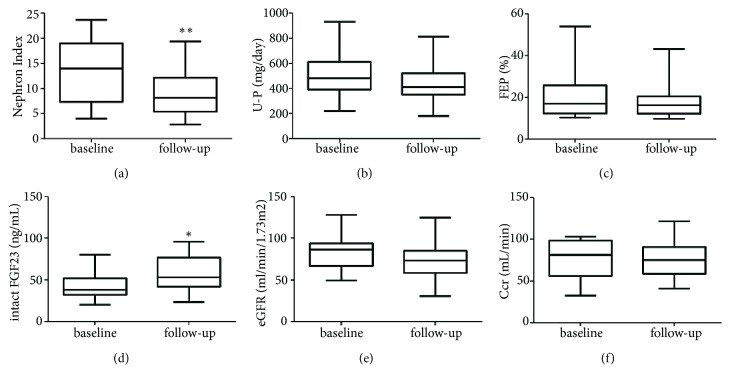
Comparisons of nephron index (a), daily (24 h) urinary phosphate excretion (U-P) (b), fractional excretion of phosphate (FEP) (c), serum intact FGF23 (d), estimated glomerular filtration rate (eGFR) (e), and creatinine clearance (Ccr) (f) between baseline and time after follow-up in the patients with type 2 diabetes. *∗p *< 0.05 vs. baseline, *∗∗p *< 0.01 vs. baseline.

The average age and follow-up period were 64 ± 8 and 5.4 ± 2.9 years, respectively (Table 1). We observed no significant changes in the eGFR at the baseline and at the follow-up (83 ± 24 mL/min/1.73 m^2^ and 75 ± 25 mL/min/1.73 m^2^,* p* = 0.082). However, we found a significant reduction in the nephron index from 13.7 ± 6.5 to 9.1 ± 4.6 (*p* = 0.001) and a significant elevation in the serum FGF23 from 43.3 ± 18.2 to 55.0 ± 20.8 ng/mL (*p* = 0.022). We did not find any change in fractional excretion of phosphate (FEP) (%) after the follow-up (21 ± 12% and 19 ± 9.0%,* p* = 0.211) (Figure 1) and in serum phosphate and calcium levels (data not shown). In addition, there was no significant change in the measured GFR (Ccr: creatinine clearance) between the baseline and the follow-up period (77.0 ± 22.9 mL/min and 73.8 ± 22.8 mL/min,* p* = 0.1999). The eGFR decline slope was −1.11 ± 2.64 mL/min/1.73 m^2^/year. During the follow-up, there were no cardiovascular events.

## 4. Discussion

In this retrospective observational study, we detected the decline in renal function during the ~5-year follow-up period using the nephron index, but not the eGFR. We propose that the nephron index may potentially be useful as a biomarker for monitoring the decline of renal function in the early stages of diabetic CKD patients.

Because the hormone FGF23 increases phosphate excretion per nephron, it is thought to correlate with FEP, which is defined as the ratio of phosphate clearance ( = (*Up* × *V*)/*Pp*) to creatinine clearance ( = (*Ucr* × *V*)/*Pcr*).

(3) $$\begin{array}{c} FEP=\frac{\left(Up\times V\right)/Pp}{\left(Ucr\times V\right)/Pcr}=\frac{Up\times V}{Ccr\times Pp} \end{array}$$

where* Up* is urinary phosphate concentration,* V* is urine volume (24 h),* Pp* is serum phosphate concentration,* Ucr* is urinary creatinine concentration,* Pcr* is serum creatinine concentration, and* Ccr* is creatinine clearance.

Higher serum FGF23 levels are, in fact, associated with higher FEP. However, in early to moderate CKD, some patients have a lower FEP than others with the same serum FGF23 level, suggesting the variability among individuals in renal sensitivity to FGF23. Those considered resistant to FGF23 because they exhibit low FEP relative to high FGF23 are associated with poor clinical outcomes [8]. In the present study, we found no change in FEP after follow-up. These results suggested the importance of considering not only FEP but also other mineral metabolic parameters in the early stages of CKD. The ratio of FEP to FGF23 may thus be regarded as a parameter of renal sensitivity to FGF23. From (2),

(4) $$\begin{array}{c} Up\times V=nephron\;\;index\times FGF23 \end{array}$$

and substitute *nephron*  *index* × *FGF*23 for *Up* × *V* in (3):

(5) FEP=nephron  index×FGF23Ccr×Pp

(6) ∴FEPFGF23=nephron  index×1Ccr×Pp

Equation (6) indicates that the renal sensitivity to FGF23 (the ratio of FEP to FGF23) correlates with the nephron number when the renal function (Ccr) and serum phosphate levels (Pp) are constant. In other words, CKD patients with reduced renal sensitivity to FGF23—probably because their residual nephron number is low—are associated with poor clinical outcomes. However, one report of moderately progressed CKD patients during 5-year follow-up found that FEP did not modify the relation between FGF23 and renal and vascular outcome [9]. In another study, a low FEP/FGF23 ratio was associated with the severity of aortic calcification in CKD stages 3–4 patients [10]. Contrastingly, Haruhara et al. revealed that the glomerular density (the number of glomeruli per total renal cortical area) evaluated by renal biopsy specimens was decreased in patients with hypertensive nephrosclerosis with a preserved eGFR compared with that in kidney transplant donors [11]. This result suggested that even if eGFR was preserved, low glomerular density is an important characteristic in hypertensive patients. Particularly, diabetic kidneys presented both glomerular changes and tubular injury before microalbuminuria or macroalbuminuria [12, 13]. We consider that diabetes decreases the number of nephrons, even when eGFR is preserved and impairs renal sensitivity to FGF23. FGF23 elevation in early CKD is an adaptive response to maintain phosphate metabolism by its phosphaturic action. Simultaneously the expression of *α*-klotho, a coreceptor for FGF23, is reduced in the kidney, leading to FGF23 hyporesponsiveness, namely, FGF23 resistance in kidney [4, 5]. Nephron index could be a novel early CKD marker and useful index for estimating FGF23 resistance in kidney.

There were some limitations of the present study. The first limitation of our study is its small number and retrospective observational nature. Large-size, prospective studies are necessary to verify whether the nephron index is a useful predictor of the decline of CKD. Second, we could not examine the pathological analysis by using human kidney specimen. Pathological examination is necessary to validate whether nephron index reflects exact nephron numbers. Finally, we enrolled only uncontrolled type 2 diabetic patients who had undergone a second admission for diabetes control. In that sense, the participants of this study were highly selected, and the follow-up period was limited. Moreover, it will be necessary to compare the present results of nephron index with those in other renal diseases, such as glomerular diseases.

In summary, the nephron index—defined as the ratio of urinary phosphate excretion to serum FGF23—holds promise as an indicator for clinical outcomes among CKD patients with similar renal function and as a noninvasive estimation of the residual nephron number. We might estimate residual nephron number by using nephron index and nephron index could be an early intervention target for CKD patients with type 2 diabetes. Further prospective and interventional studies using the nephron index are essential to establish its usefulness in the management of CKD patients.

## Figures and Tables

**Table 1:** Baseline characteristics of patients.

Variables and parameters	Baseline
Age (years)	64 ± 8
Sex, male (%)	4 (27)
BMI (kg/m^2^)	26.5 ± 3.3
Duration of diabetes (years)	11 ± 6.2
Diabetic retinopathy, n (%)	9 (60)
Dyslipidemia, n (%)	14 (93)
Hypertension, n (%)	10 (67)
HbA1c (%)	10.3 ± 2.2
HbA1c (mmol/L)	89 ± 24.5
BUN (mg/dL)	14 ± 3.1
Cr (mg/dL)	0.59 [0.50–0.74]
eGFR (mL/min/1.73 m^2^)	83 ± 24
Serum magnesium (mg/dL)	1.9 [1.75–2.00]
Serum phosphate (mg/dL)	3.5 ± 0.60
Creatinine clearance (mL/min)	77 ± 23
Urinary albumin excretion (mg/day)	11 (7.9–34)
Urinary phosphate excretion (mg/day)	511 ± 176

Data are expressed as means ± standard deviation (SD), and skewed variables are described as medians with an interquartile range. BMI, body mass index; HbA1c, glycated haemoglobin; BUN, blood urea nitrogen; Cr, creatinine; eGFR, estimated glomerular filtration rate.

## Data Availability

The original data used to support the findings of this study are available from the corresponding author upon request.
